# Editorial for the Special Issue on Broadband Terahertz Devices and Communication Technologies

**DOI:** 10.3390/mi14051044

**Published:** 2023-05-12

**Authors:** Lu Zhang, Xiaodan Pang, Prakash Pitchappa

**Affiliations:** 1 College of Information Science and Electronic Engineering, Zhejiang University, Hangzhou 310027, China; 2 Department of Applied Physics, KTH Royal Institute of Technology, 114 19 Stockholm, Sweden; 3 Institute of Microelectronics, Agency for Science, Technology and Research, Singapore 138634, Singapore

The remarkable explosion of wireless devices and bandwidth-consuming Internet applications have boosted the demand for wireless communications with ultra-high data rate. The wireless traffic volume is foreseen to match or even surpass the wired services by 2030, and high-precision wireless services will need to be guaranteed with a peak data rate of well beyond 100 Gbit/s, eventually reaching 1 Tbit/s. To meet the exponentially increasing traffic demand, new regions in the radio spectrum are being explored. The terahertz band, which is sandwiched between microwave frequencies and optical frequencies, is considered a next breakthrough point to revolutionize communication technology due to its rich spectrum resources. It is recognized as a promising candidate for future rate-greedy applications, such as 6G communications. At the World Radio Communication Conference 2019 (WRC-19), it was announced that the identification of frequency bands in the frequency range of 275 GHz–450 GHz is permitted for land-mobile and fixed service applications, indicating potential standardization of the low-frequency window of terahertz band for near-future wireless communications.

Motivated by the potential of terahertz wireless communications, this Special Issue reports on recent critical technological breakthroughs in terms of broadband terahertz devices and communications, as well as novel technologies at other frequency bands that can also motivate terahertz research. Five studies [1,2,3,4,5] present key devices for terahertz communications, including terahertz reconfigurable intelligent surfaces [1], terahertz micro-electro-mechanical system (MEMS) switches [2], resonant triple-band terahertz thermal detectors [3], G-band continuous-wave traveling wave tubes [4], and wide-dynamic-range GaAs transceivers [5], which could effectively support broadband terahertz systems. Furthermore, we have also selected three interesting research studies [6,7,8] on low-frequency bands for this Special Issue, including the design of 5G multiple-input multiple-output (MIMO) antennas [6,7] and differential low-noise amplifiers [8]. We believe these works could also motivate research on terahertz communication devices and systems for 6G communications and other typical application scenarios. With the advances in broadband terahertz devices and the design of novel digital signal-processing routines, high-speed terahertz communications could be realized. In this Special Issue, three terahertz communicating systems were analyzed and demonstrated [9,10,11], including a 144 Gbps photonics terahertz communication system working at 500 GHz [9], a W-band communication and sensing convergence system [10], and an analysis of secure terahertz communications with perfect electric conductor (PEC) and multiple eavesdroppers [11].

To overcome the high loss and line-of-sight connectivity challenges of terahertz communication links, reconfigurable intelligent surfaces (RISs) are widely analyzed. However, active elements used for 5G RIS are often impractical for future 6G communications due to cutoff frequency limitations and higher loss at terahertz frequencies. Yang et al. [1] provided a comprehensive review on reconfigurable metasurfaces operating in the terahertz band with the potential to assist 6G communication links, categorized based on tuning mechanisms, including complementary metal-oxide-semiconductor (CMOS) transistors, Schottky diodes, high-electron mobility transistors (HEMTs), graphene, photoactive semiconductor materials, phase-change materials (vanadium dioxide, chalcogenides, and liquid crystals), and MEMS. Terahertz RISs are believed to be crucial for actualizing 6G communication links.

MEMS switches are important elements for future terahertz communication networks, but designing them for the terahertz frequency band is challenging since their physical dimensions are comparable to the wavelength. Feng et al. [2] designed and realized a terahertz MEMS switch using both silicon and fused quartz as examples of high and low dielectric-constant substrates, respectively. Both silicon and fused-quartz switches were calibrated based on the two-port through-reflection-line method from 140 to 750 GHz. At 750 GHz, the measurement results from the switches on both substrates show an ON-state insertion loss of less than 3 dB and an OFF-state isolation greater than 12 dB.

Terahertz waves possess unique properties, such as the ability to penetrate non-conductive materials and identify specific materials based on their characteristic terahertz signatures. Thus, terahertz detectors show great application potential in imaging, spectroscopy, and sensing fields. Wang et al. [3] experimentally validated a room-temperature CMOS monolithic resonant triple-band terahertz thermal detector. The responsivity, noise equivalent power, and thermal time constant of the detector were experimentally assessed at 0.91 THz, 2.58 THz, and 4.2 THz. The detector also has natural scalability to focal plane arrays, demonstrating significant advances in developing compact, room-temperature, low-cost, and mass-production multiband terahertz detection systems.

At the terahertz band, the G-band electromagnetic wave provides availability for the design of terrestrial and satellite radio communication networks according to the radio regulations of the International Telecommunication Union. The European Commission Horizon 2020 ULTRAWAVE project aims to exploit portions of the millimeter-wave spectrum for creating a very high-capacity layer. Feng et al. [4] presented the development of a G-band broadband continuous-wave traveling wave tube for wireless communications based on a slow-wave structure of fold waveguide. The device successfully provides a saturation output power over 8 W and a saturation gain over 30.5 dB with a bandwidth of 27 GHz.

Due to the abundance of spectrum resources in the millimeter-wave band, the WRC-19 conference approved multiple mm-wave spectra for future mobile communication research and development, including the 66–67 GHz frequency range, which is near the terahertz band. Zhou et al. [5] presented a 66–67 GHz transceiver monolithic microwave-integrated circuit (MMIC) in a waveguide module for massive MIMO channel emulator applications. The proposed transceiver integrates a tripler chain for local oscillator drive, a mixer, and a band-pass filter using a 0.1 µm pHEMT GaAs process. A high dynamic output power range, up to 50 dB over 66-to-76 GHz, is achieved by dealing with all unwanted harmonic signals employing highly selective band-pass and high-pass filters in the transceiver. The total power consumption of the chip is 645 mW with a supply voltage of 5 V.

In addition to the aforementioned research works, we have also selected three interesting studies [6,7,8] at low-frequency bands for this Special Issue, including the design of 5G MIMO antennas by Sheriff et al. [6] and Zhang et al. [7], and differential low-noise amplifiers by Wang et al. [8]. We believe these studies could also motivate research on terahertz communication devices and systems for 6G communications and other typical application scenarios.

Supported by broadband terahertz devices, terahertz communication systems have developed rapidly in recent years. Terahertz wireless communication systems based on photonics have emerged as promising candidates for 6G communications, capable of providing hundreds of Gbps or even Tbps data capacity. Liu et al. [9] experimentally demonstrated a 144 Gbps dual-polarization quadrature-phase-shift-keying (DP-QPSK) signal generation and transmission over a 20 km SSMF and 3 m wireless 2 × 2 MIMO link. A novel and low-complexity joint deep belief network (J-DBN) equalizer was proposed to compensate for linear and nonlinear distortions during fiber–wireless transmission. This scheme shows promises for meeting future fiber–terahertz integration communication demands for low power consumption, low cost, and high capacity.

The convergence of communication and sensing is highly desirable for future wireless systems. Idrees et al. [10] presented a converged system using a single orthogonal frequency-division multiplexing (OFDM) waveform and proposed a novel method, based on the zero-delay shift for received echoes, to extend the sensing range beyond the cyclic prefix interval (CPI). Both simulation and proof-of-concept experiments evaluated the performance of the proposed system at the W-band (97 GHz). The experiment employed a W-band heterodyne structure to transmit/receive an OFDM waveform featuring 3.9 GHz bandwidth with quadrature amplitude modulation (16-QAM). The proposed approach successfully achieves a range resolution of 0.042 m and a speed resolution of 0.79 m/s with an extended range, revealing the potential of terahertz technologies in the field of communication and sensing convergence.

The transmission security of high-speed THz wireless links is an important issue in terahertz research. He et al. [11] comprehensively investigated the physical layer security issue of a terahertz communication system in the presence of multiple eavesdroppers and beam scattering. The method of moments (MoM) was adopted to characterize the eavesdroppers’ channel. To establish a secure link, traditional beamforming and artificial noise (AN) beamforming were considered as transmission schemes for comparison. The numerical results show that eavesdroppers can indeed degrade the secrecy performance by changing the size or location of the PEC, while the AN beamforming technique can be an effective candidate to counterbalance this adverse effect.

We would like to thank all the authors for their submissions to this Special Issue. We also thank the reviewers for dedicating their time and helping to ensure the quality of the submitted papers. Finally, we are grateful to the staff at the Editorial Office of *Micromachines*, and in particular Mr. Scott Wang, for their efficient assistance.
